# Planetary Perihelia of 1881

**Published:** 1881-02

**Authors:** 


Planetary Perihelia of 1881.—Whether astrologers are right in their prognostications of remarkable phenomena occurring as a consequence of the perihelia of certain planets in 1881, or not, it is evident to most of our senses that the winter thus far has been a very severe one in the amount of snow and reduced temperature we have experienced. That the unusual relations of some of the planets to each other, and to our earth, which are to occur in 1881, may so disturb the equilibrium of our usual electrical state, or condition, as to develop new or infrequent forms of disease is quite probable; but, could we be certain of such results, it is not quite clear that any useful prophylactic or precautionary measures could be used for preventing them, or for protecting ourselves against such visitations. Under these circumstances we will be compelled to bear up against these calamities with such philosophy as we might be able to command. However, Mr. Vennor has not seen fit to enlighten us on all the points and we will await developments. Nous Verrons.

